# Association between gut microbial diversity and technique failure in peritoneal dialysis patients

**DOI:** 10.1080/0886022X.2023.2195014

**Published:** 2023-04-03

**Authors:** Shulan Guo, Huan Wu, Ji Ji, Zhaoxing Sun, Bo Xiang, Weiwei Wu, Jun Ji, Jie Teng, Xiaoqiang Ding, Xiaofang Yu

**Affiliations:** a Department of Nephrology, Zhongshan Hospital, Fudan University, Shanghai, China; b Shanghai Medical Center of Kidney, Shanghai, China; c Shanghai Key Laboratory of Kidney and Blood Purification, Shanghai, China; d Shanghai Institute of Kidney and Dialysis, Shanghai, China; e Department of Nephrology, Xiamen Branch, Zhongshan Hospital, Fudan University, Xiamen, China; f Xiamen Clinical Quality Control Center of Nephrology, Xiamen, China

**Keywords:** Peritoneal dialysis, technique failure, gut dysbiosis, gut microbial diversity, uremic toxins

## Abstract

**Background:**

Gut dysbiosis in peritoneal dialysis (PD) patients causes chronic inflammation and metabolic disorders which result in a series of complications, probably playing an important role in PD technique failure. The reduction in gut microbial diversity was a common feature of gut dysbiosis. The objective was to explore the relationship between gut microbial diversity and technique failure in PD patients.

**Methods:**

The gut microbiota was analyzed by 16s ribosomal RNA gene amplicon sequencing. Cox proportional hazards models were used to identify association between gut microbial diversity and technique failure in PD patients.

**Results:**

In this study, a total of 101 PD patients were enrolled. During a median follow-up of 38 months, we found that lower diversity was independently associated with a higher risk of technique failure (hazard ratio [HR], 2.682; 95% confidence interval [CI], 1.319–5.456; *p* = 0.006). In addition, older age (HR, 1.034; 95% CI, 1.005–1.063; *p* = 0.020) and the history of diabetes (HR, 5.547; 95% CI, 2.218–13.876; *p* < 0.001) were also independent predictors for technique failure of PD patients. The prediction model constructed on the basis of three independent risk factors above performed well in predicting technique failure at 36 and 48 months (36 months: area under the curve [AUC] = 0.861; 95% CI, 0.836–0.886; 48 months: AUC = 0.815; 95% CI, 0.774–0.857).

**Conclusion:**

Gut microbial diversity was independently correlated with technique failure in PD patients, and some specific microbial taxa may serve as a potential therapeutic target for decreasing PD technique failure.

## Introduction

More and more patients with end-stage kidney disease (ESKD) prefer to choose peritoneal dialysis (PD) as the first kidney replacement therapy (KRT) [1,2]. Compared with hemodialysis (HD), PD has many advantages, including lower cost, improved quality of life, preservation of residual kidney function (RKF) and less changes in hemodynamics [3–6]. However, with the extension of the PD treatment time, most PD patients lack the opportunities for kidney transplantation due to the shortage of organ donors, and often transfer to HD or directly die because of peritonitis, inadequate dialysis or other complications of PD [7,8]. These poor prognoses are collectively called PD technique failure which limits PD patients to stay on PD treatment for a long time, so it’s critical to identify factors associated with the technique failure and offer appropriate preventive strategies.

Gut microbiota is demonstrated to be related to the well-being of hosts [9], and a healthy gut microbial community is generally characterized by high taxa diversity, high microbial gene richness and stable microbiome functional cores [10]. Gut dysbiosis has been reported in numerous chronic diseases including ESKD [11–14]. In addition to the kidney diseases themselves, PD also contributes to gut dysbiosis because of long dialysis duration, high peritoneal glucose exposure and loss of RKF [15]. We and other researchers found that PD patients exhibited lower alpha diversity and increased abundance of opportunistic pathogenic bacteria compared to healthy controls [16,17]. In turn, aberrant gut microbiota causes inflammation and metabolic disorders in PD patients and generates more uremic toxins which aggravate clinical symptoms and outcomes [18].

Declined gut microbial diversity is a simple but useful indicator of gut dysbiosis and disease development. Peled et al. [19] indicated that higher intestinal diversity was associated with a lower risk of death after allogeneic hematopoietic-cell transplantation. Lin et al. [20] also found that HD patients with lower microbial diversity had a higher risk of cardiovascular events and death from any cause. However, it is unclear whether decreased gut microbial diversity is still associated with adverse outcomes in PD patients. In this study, we aimed to explore the relationship between gut microbial diversity and technique failure in PD patients.

## Materials and methods

### Study design and population

This was a prospective cohort study conducted in the peritoneal dialysis center, Zhongshan Hospital, Fudan University. The study design and patients were previously described [16]. Briefly, patients who had been on continuous ambulatory peritoneal dialysis (CAPD) treatment for at least 6 months were recruited from February 2018 to July 2019. Patients were excluded if they had gastrointestinal diseases, severe liver diseases, tumor, other immunological or autoimmune disorders or had used any specific drugs in the previous 3 months, including antibiotics, probiotics, prebiotics, synbiotics, proton pump inhibitors and immunosuppressive agents. Those younger than 18 years old or non-Han nationality were also excluded. All patients were using glucose dialysate of Baxter. The study was approved by the Ethical Committee of Zhongshan Hospital, Fudan University (Approval No.: B2017–108R). All participants provided written informed consent.

### Fecal and serum samples collection

Fecal samples were collected in sterile plastic pots by patients at home, delivered to our nephropathy laboratory on dry ice, aliquoted to 200 mg subsamples, and immediately stored at −80 °C for further analysis. Venous blood samples were obtained in the morning and under fasting conditions. After centrifugation (3000×*g*, 10 min), the serum samples were isolated and immediately frozen at −80 °C until use.

### Data collection

Baseline demographic characteristics were documented at the time of collecting samples, including age, gender, height, weight, blood pressure (BP), urine volume, dialysis prescription, underling kidney disease, dialysis duration, comorbidities, prior peritoneal dialysis-associated peritonitis episodes, and history of taking medicine. Height and weight were measured while patients wore light clothes without shoes, and body mass index (BMI) was calculated as the dry weight in kilograms divided by the height in meters squared. According to the dialysis prescription, glucose exposure was calculated as the product of the glucose concentration times the volume for each exchange [21]. Comorbidities included hypertension, diabetes, coronary heart disease and cerebral infarction. All drugs taken within 3 months before fecal samples collecting were recorded. Questionnaires were administered to PD patients to collect information about the number of bowel movements per week and the Bristol Stool Form Scale.

The biochemical parameters were measured using standard methods followed in Zhongshan Hospital, such as hemoglobin, serum creatinine, blood urea nitrogen (BUN), uric acid, albumin, triglyceride, total cholesterol, calcium, phosphorus, high-sensitivity C-reactive protein (hsCRP) and so on. Indoxyl sulfate (IS), p − cresyl sulfate (PCS) and trimethylamine N-oxide (TMAO) levels were detected using high performance liquid chromatography mass spectrometry (HPLC-MS) as previously described [22]. Normalized protein nitrogen appearance rate (nPNA) was calculated by Randerson’s equation [23]. A standard peritoneal equilibration test (PET) was performed shortly before or after sample collection, and weekly Kt/V (kidney/peritoneum/total) were calculated by using PD adequest 2.0 software (Baxter Healthcare Corporation, Norfolk, UK) [24].

### 16S rRNA microbial profiling analysis

Bacterial DNA was extracted from stool sample using The E.Z.N.A.^®^ Stool DNA Kit (Omega Bio-tek, Inc., GA) according to the manufacturer’s instructions. The V3∼V4 region of 16S rRNA genes was amplified by polymerase chain reaction with the primers F1 and R2 (5′-CCTACGGGNGGCWGCAG-3′ and 5′-GACTACHVGGGTATCTAATCC-3′) [25]. The products from different samples were indexed and mixed at equal ratios for sequencing by Shanghai Mobio Biomedical Technology Co. Ltd. using the Miseq platform (Illumina Inc., USA). Those quality-filtered sequences extracted from raw data using USEARCH (version 11.0.667) were clustered into unique sequences and sorted in order of decreasing abundance to identify representative sequences using UPARSE according to UPARSE OTU analysis pipeline [26], and singletons were omitted in this step. Operational Taxonomic Units (OTUs) were classified based on 97% similarity after chimeric sequences were removed using UPARSE (version 7.1 http://drive5.com/uparse/) and were annotated using the SILVA reference database (SSU138) in qiime2–2020.11 [27].

Alpha diversity was assessed by using Mothur v1.42.1 [28] and presented as the inverse Simpson index. Bray-Curtis was calculated in QIIME (v1.9.1) [29] for evaluating beta diversity. Principal coordinate analysis (PCoA) plots which were used to test for statistical significance between the groups using 10,000 permutations were generated in R (version 3.6.0) package vegan 2.5–7. The linear discriminant analysis (LDA) effect size (LEfSe) was used to detect taxa with differential abundance among groups [30], and only taxa with LDA score > 2.5 were presented.

### Study outcome

The primary outcome of this study was PD technique failure, defined as transfer to HD for more than 30 days, or death while on PD therapy or within 30 days of transfer from PD to HD [31]. All patients were followed up until transfer to HD, death, kidney transplantation or at the end of follow-up on 30 June 2022.

### Statistical analysis

Patients were stratified into higher-diversity and lower-diversity groups according to the median alpha diversity value, which we calculated using the inverse Simpson index. Continuous variables were expressed as means ± standard deviations (SDs) for normal distributions or medians and interquartile ranges (IQRs) for non-normal distributions, and categorical variables were expressed as frequencies and percentages. To compare the baseline characteristics between two groups, the Student’s *t*-test or Mann–Whitney U test was used for continuous data, and the Chi-squared test or Fisher exact test was used for categorical data. The correlations between alpha diversity and clinical variables were analyzed by Spearman rank correlation analysis. Peritonitis rates were expressed as episodes per patient-year [32] and compared using Poisson regression. The Kaplan–Meier method was applied to estimate and plot survival curves of technique survival, and the Log-rank test was used to assess the differences between two groups. Univariate Cox proportional hazards analysis was performed to identify factors associated with the technique failure, and then significant variables (*p* < 0.05) in univariate analyses were added to multivariate Cox proportional hazards model for adjustment. The results were shown as the hazard ratios (HRs) and 95% confidence intervals (CIs). The time-dependent receiver operating characteristic (ROC) curves were used to analyze the predictive power of model which was comprised of the independent risk factors. A two-tailed *p* value <0.05 was considered statistically significant. All analyses were performed using SPSS (version 23.0), GraphPad Prism (version 9.0), Stata (version 15.1) or R (version 4.1.0).

## Results

### Patient characteristics

**Figure 1.**
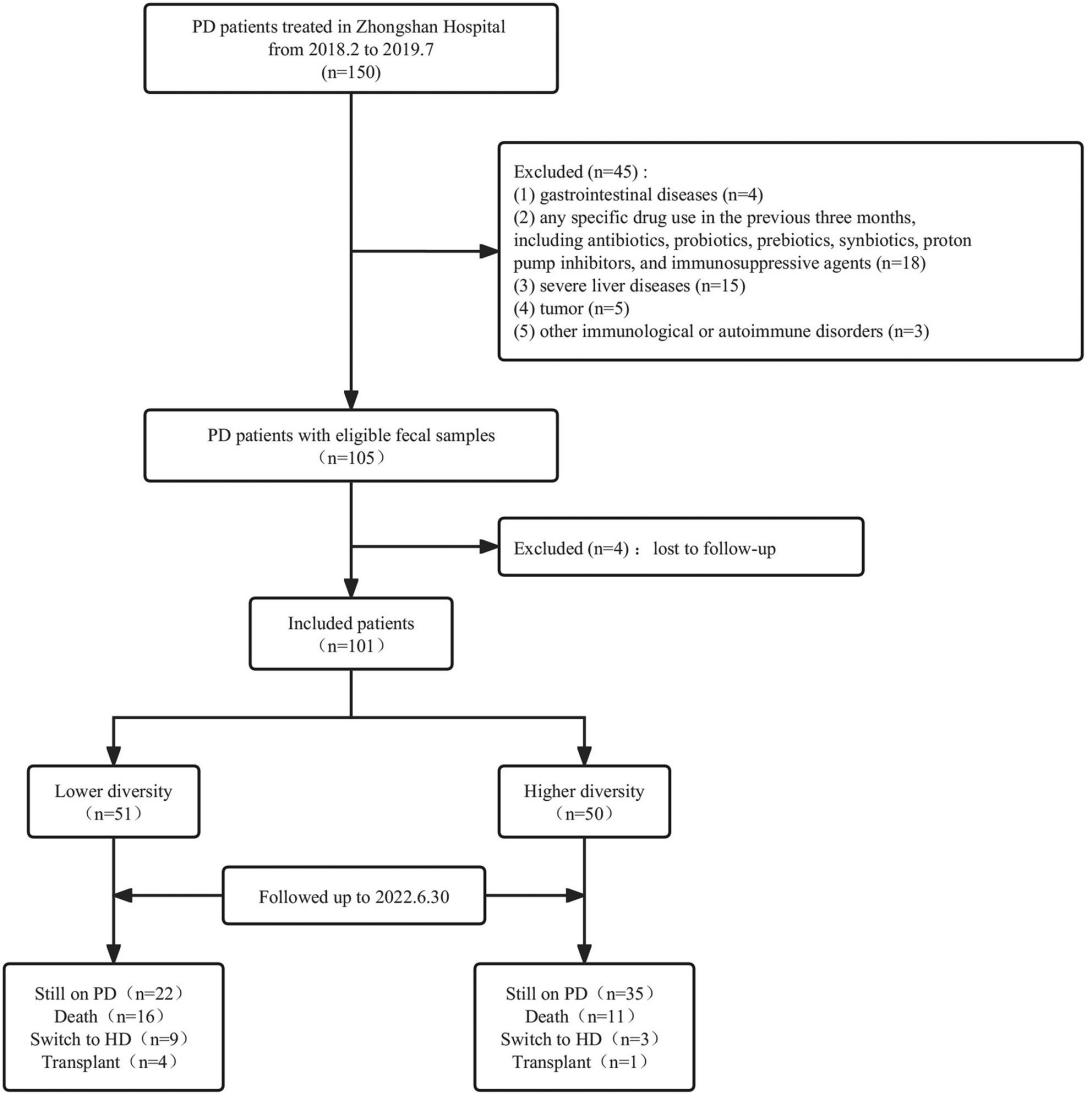
Flow chart of the study. PD: peritoneal dialysis; HD: hemodialysis.

**Figure 2.**
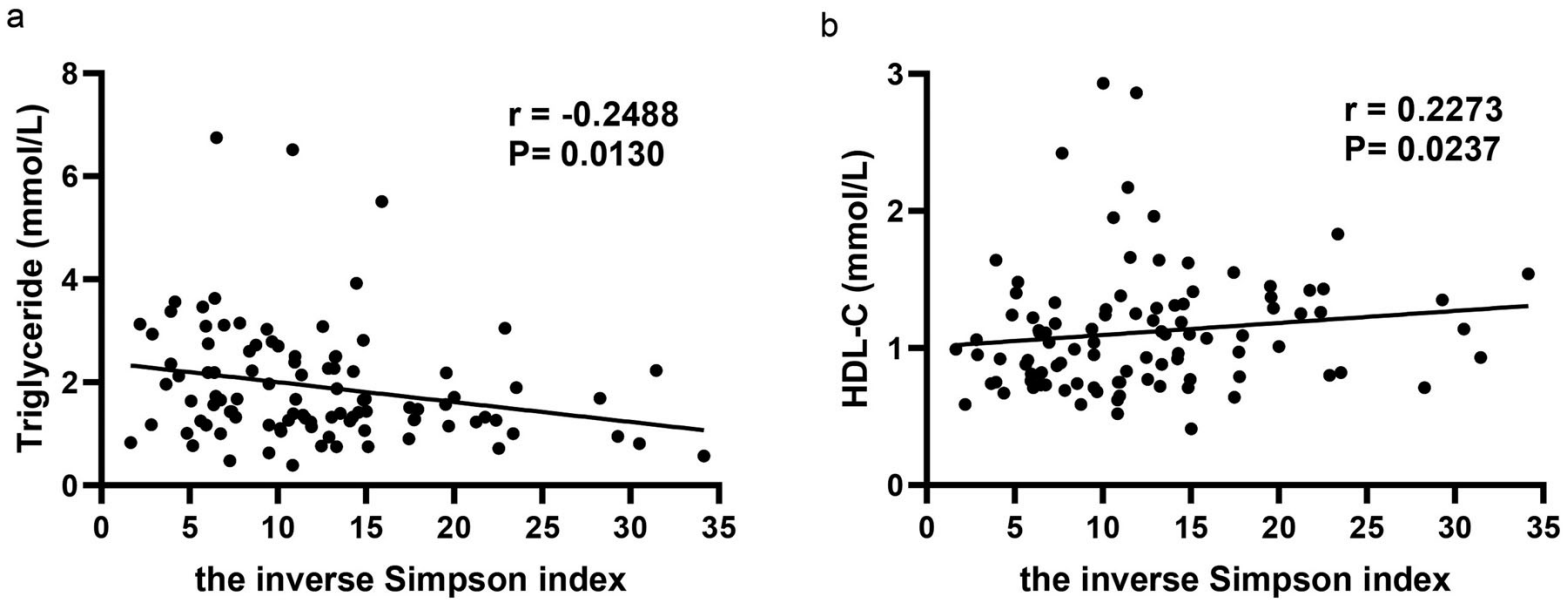
Factors associated with the inverse Simpson index. (a) Triglyceride, (b) HDL-C. HDL-C: high-density lipoprotein cholesterol.

We collected eligible fecal samples from 105 CAPD patients between February 2018 and July 2019. Among these, four patients were excluded because they were only hospitalized in our PD center occasionally and lost to follow-up after sample collecting. Finally, a total of 101 patients were enrolled in this study (Figure 1). As shown in Table 1, there were 48 males (47.5%) and 53 females (52.5%), with an average age of 57.07 ± 13.74 years old and a median dialysis duration of 21 (IQR, 12–29.5) months. The causes of ESKD mainly included primary glomerular disease (39.6%), diabetic kidney disease (13.9%), hypertensive kidney disease (8.9%), polycystic kidney disease (3%) and others (5.9%). The proportions of patients coexistent with hypertension, diabetes, cerebral infarction and coronary heart disease were 95%, 22.8%, 10.9%, and 5% respectively. According to the median (11.35) of inverse Simpson index reflecting species diversity, richness and evenness of gut microbiota, patients were stratified into lower-diversity group (≤11.35) and higher-diversity group (>11.35). Compared with the higher-diversity group, patients in the lower-diversity group had higher triglyceride and lower high-density lipoprotein cholesterol (HDL-C) (*p* < 0.05), with no significant differences in other baseline characteristics. Tables 1 and 2 presented the comparisons of demographic and biochemical characteristics between the two groups. As a continuous variable, the inverse Simpson index was significantly correlated with triglyceride (*r* = −0.249, *p* = 0.013) and HDL-C (*r* = 0.227, *p* = 0.024) (Figure 2). The associations were not detected between remaining clinical variables and microbial diversity (Supplementary Table 1).

**Table 1.** Baseline demographic and clinical characteristics of PD patients.

Characteristics	All patients (*n* = 101)	Lower diversity (≤11.35, *n* = 51)	Higher diversity (>11.35, *n* = 50)	*p* Value
Age (years)	57.07 ± 13.74	56.04 ± 13.96	58.12 ± 13.56	0.449
Men (%)	48 (47.5%)	24 (47.1%)	24 (48%)	0.925
Systolic BP (mmHg)	139.48 ± 18.58	140.43 ± 19.66	138.50 ± 17.55	0.604
Diastolic BP (mmHg)	80.87 ± 11.19	81.43 ± 10.84	80.30 ± 11.61	0.614
Body mass index (kg/m^2^)	23.02 ± 4.22	23.63 ± 4.30	22.40 ± 4.09	0.143
Dialysis duration (months)	21 (12, 29.5)	20 (12, 28)	21 (12, 32.5)	0.747
Urine volume (L/24 h)	0.60 (0, 1.10)	0.60 (0, 1.20)	0.53 (0, 1.03)	0.451
Glucose exposure (g/day)	120.0 (90.0, 140.0)	120.0 (90.0, 140.0)	120.0 (90.0, 152.5)	0.939
Underlying kidney disease (%)				0.944
Primary glomerular disease	40 (39.6%)	20 (39.2%)	20 (40%)	
Diabetic kidney disease	14 (13.9%)	6 (11.8%)	8 (16%)	
Hypertensive kidney disease	9 (8.9%)	5 (9.8%)	4 (8%)	
Polycystic kidney disease	3 (3%)	1 (2%)	2 (4%)	
Others	6 (5.9%)	4 (7.8%)	2 (4%)	
Unknown	29 (28.7%)	15 (29.4%)	14 (28%)	
Comorbidity (%)				
Hypertension	96 (95%)	48 (94.1%)	48 (96%)	1
Coronary heart disease	5 (5%)	2 (3.9%)	3 (6%)	0.678
Diabetes	23 (22.8%)	11 (21.6%)	12 (24%)	0.771
Cerebral infarction	11 (10.9%)	6 (11.8%)	5 (10%)	0.776
Medications (%)				
ACEI	12 (11.9%)	7 (13.7%)	5 (10%)	0.563
ARB	51 (50.5%)	27 (52.9%)	24 (48%)	0.619
β-Blockers	58 (57.4%)	32 (62.7%)	26 (52%)	0.275
α-Blockers	14 (13.9%)	6 (11.8%)	8 (16%)	0.538
Antiplatelet drugs	16 (15.8%)	7 (13.7%)	9 (18%)	0.556
Statins	29 (28.7%)	18 (35.3%)	11 (22%)	0.140
Iron supplements	57 (56.4%)	29 (56.9%)	28 (56%)	0.930
Phosphate binders	57 (56.4%)	27 (52.9%)	30 (60%)	0.474
Prior peritonitis episodes (%)	12 (11.9%)	8 (15.7%)	4 (8%)	0.233
Bristol Stool Form Scale	4 (3, 5)	4 (3, 4)	4 (3.75, 5)	0.066
Number of weekly bowel movements	12 (7, 14)	12 (10, 14)	10 (7, 14)	0.552

PD: peritoneal dialysis; BP: blood pressure; ACEI: angiotensin-converting enzyme inhibitors; ARB: angiotensin receptor blockers. Data were expressed as mean ± SD, median (interquartile range) or *n* (%) as appropriate. Differences between two groups were evaluated using the Student’s *t*-test or Mann–Whitney U test for continuous data and the Chi-squared test or Fisher exact test for categorical data.

**Table 2.** Baseline biochemical characteristics of PD patients.

Characteristics	All patients (*n* = 101)	Lower diversity (≤11.35, *n* = 51)	Higher diversity (>11.35, *n* = 50)	*p* Value
Hemoglobin (g/L)	106 (93, 120.5)	107 (89, 121)	105.5 (96.8, 119.5)	0.868
Albumin (g/L)	34.93 ± 4.95	34.77 ± 4.97	35.10 ± 4.97	0.735
Prealbumin (g/L)	0.32 ± 0.06	0.31 ± 0.06	0.32 ± 0.06	0.363
BUN (mmol/L)	18.53 ± 4.87	18.53 ± 5.37	18.53 ± 4.35	0.993
Creatinine (µmol/L)	786 (638.5, 1060.5)	786 (627, 1056)	803 (644.8, 1077.5)	0.836
Uric acid (µmol/L)	373 (311, 431)	373 (311, 443)	372 (311, 416.5)	0.499
Total cholesterol (mmol/L)	4.40 ± 1.14	4.35 ± 0.94	4.45 ± 1.34	0.657
Triglyceride (mmol/L)	1.64 (1.18, 2.49)	1.97 (1.25, 2.79)	1.38 (1.14, 2.11)	0.021
LDL-C (mmol/L)	2.44 (1.87, 3.14)	2.55 (1.86, 3.01)	2.42 (1.92, 3.27)	0.622
HDL-C (mmol/L)	1.04 (0.77, 1.31)	0.91 (0.74, 1.18)	1.17 (0.92, 1.42)	0.003
Calcium (mmol/L)	2.33 (2.17, 2.40)	2.32 (2.20, 2.39)	2.34 (2.15, 2.45)	0.693
Phosphorus (mmol/L)	1.58 ± 0.45	1.62 ± 0.44	1.54 ± 0.45	0.372
iPTH (pg/mL)	133 (75.45, 275.05)	133 (76.30, 244)	136.25 (74.53, 312.48)	0.639
NT-proBNP (pg/mL)	2083 (884.1, 6402.5)	1550 (736.4, 5530)	2333.5 (1206.8, 8994)	0.209
Glucose (mmol/L)	5.10 (4.70, 5.80)	5.20 (4.40, 6.00)	5.05 (4.70, 5.73)	0.927
Total bilirubin (µmol/L)	4.70 (3.90, 6.40)	4.70 (3.80, 6.20)	4.75 (3.90, 6.80)	0.423
ALT (U/L)	14 (9, 22)	11 (8, 21)	16 (10, 22.3)	0.094
AST (U/L)	16 (11, 21.5)	14 (11, 19)	17.5 (12, 24.3)	0.066
ALP (U/L)	71 (53, 86.5)	69 (51, 82)	72 (57, 96.8)	0.244
hsCRP (mg/L)	2.80 (0.90, 7.58)	2.85 (0.90, 7.40)	2.50 (0.83, 9.75)	0.974
nPNA (g/kg/d)	0.94 (0.76, 1.09)	0.94 (0.76, 1.09)	0.95 (0.76, 1.09)	0.514
Kt/V (total)	2.04 (1.72, 2.40)	2.02 (1.72, 2.39)	2.08 (1.69, 2.41)	0.548
Kt/V (kidney)	0.48 (0, 1.09)	0.59 (0, 1.07)	0.31 (0, 1.14)	0.598
Kt/V (peritoneum)	1.53 ± 0.42	1.48 ± 0.45	1.57 ± 0.40	0.309
Peritoneal transport type (%)				0.243
Low	4 (4%)	3 (5.9%)	1 (2%)	
Low average	38 (37.6%)	23 (45.1%)	15 (30%)	
High average	49 (48.5%)	20 (39.2%)	29 (58%)	
High	10 (9.9%)	5 (9.8%)	5 (10%)	
IS (µg/L)	25.30 (12.80, 37.10)	25.40 (12.30, 38.40)	25.20 (16.25, 37.10)	0.860
PCS (µg/L)	18.35 (8.70, 30.83)	18.70 (8.77, 30.60)	18 (8.00, 31.15)	0.931
TMAO (µg/L)	4.09 (2.96, 6.43)	4.09 (2.80, 7.05)	3.98 (3.00, 5.62)	0.783

PD: peritoneal dialysis; BUN: blood urea nitrogen; LDL-C: low-density lipoprotein cholesterol; HDL-C: high-density lipoprotein cholesterol; iPTH: intact parathyroid hormone; NT-proBNP: N-terminal pro-brain natriuretic peptide; ALT: alanine aminotransferase; AST: aspartate aminotransferase; ALP: alkaline phosphatase; hsCRP: high-sensitivity C-reactive protein; nPNA: normalized protein nitrogen appearance rate; IS: indoxyl sulfate; PCS: p-cresyl sulfate; TMAO: trimethylamine N-oxide. Data were expressed as mean ± SD, median (interquartile range) or *n* (%) as appropriate. Differences between two groups were evaluated using the Student’s *t*-test or Mann–Whitney U test for continuous data and the Fisher exact test for categorical data.

**Figure 3.**
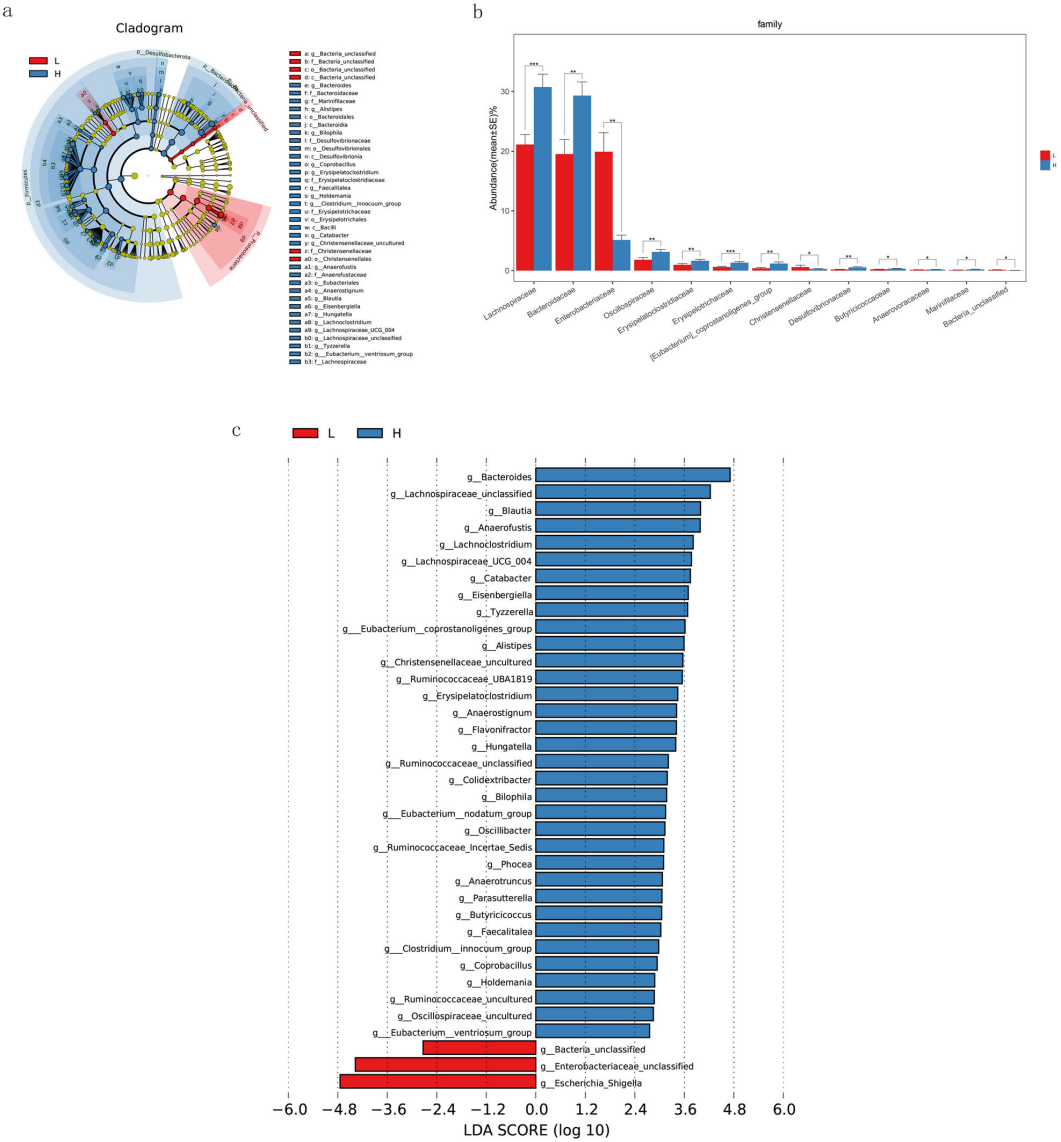
Alteration of the gut microbial taxa exhibited by the patients in the lower- and higher-diversity groups across different taxonomic levels. (a) Cladogram showing differentially abundant taxa of the gut microbiota across different taxonomic levels. (b) The microbial taxa with significantly different abundance between two groups at the family level. (c) The significantly different taxa between two groups at the genus level. *p < 0.05, **p < 0.01, ***p < 0.001. LDA: linear discriminant analysis; L: lower diversity; H: higher diversity.

A significant difference was observed in the gut microbial composition between the two groups (*p* = 0.001) (Supplementary Figure 1). The taxa with differential abundance between the two groups across different taxonomic levels were shown in Figure 3. At the family level, the relative abundance of Enterobacteriaceae was significantly increased in PD patients with lower diversity compared with those with higher diversity (*p* < 0.01). At the genus level, three genera including Escherichia Shigella, Enterobacteriaceae-unclassified and Bacteria-unclassified were significantly enriched in lower-diversity group, while patients in higher-diversity group had a higher expression of 34 genera including Bacteroides, Lachnospiraceae-unclassified, Blautia and so on (LDA score > 2.5).

### Technique survival analysis

During a median follow-up of 38 months (IQR, 31–44.5), 57 CAPD patients (56.4%) were still on PD treatment, 5 (5%) accepted kidney transplantation, 12 (11.9%) transferred to HD treatment, and 27 (26.7%) occurred death. The reasons of switching to HD included inadequate dialysis, infection and mechanical reasons (Supplementary Table 2). Overall, 39 (38.6%) PD technique failure were recorded (Table 3). In addition, a total of 52 episodes of peritonitis occurred in 29 patients (0.18 episodes per patient-year), and there was no significant difference in the peritonitis rates between the lower- and higher-diversity groups (0.20 episodes per patient-year vs. 0.15 episodes per patient-year, *p* = 0.254).

**Table 3.** Clinical outcomes of PD patients.

	All patients (*n* = 101)	Lower diversity (≤11.35, *n* = 51)	Higher diversity (>11.35, *n* = 50)	*p* Value
Follow-up time (months)	38 (31, 44.5)	35 (30, 43)	39 (34, 48)	0.047
Total episodes of peritonitis	52	29	23	–
Time at risk (patient-years)	296.67	141.92	154.75	–
Peritonitis rate (episodes per patient-year)	0.18	0.20	0.15	0.254
Outcomes				0.014
Technique failure (%)	39 (38.6%)	25 (49%)	14 (28%)	
Death	27 (26.7%)	16 (31.4%)	11 (22%)	
Switch to HD	12 (11.9%)	9 (17.6%)	3 (6%)	
Kidney transplantation (%)	5 (5%)	4 (7.8%)	1 (2%)	
Still on PD (%)	57 (56.4%)	22 (43.1%)	35 (70%)	

PD: peritoneal dialysis; HD: hemodialysis. Peritonitis rates were expressed as episodes per patient-year and compared using Poisson regression. Remaining data were expressed as median (interquartile range) or n (%) as appropriate. Differences between two groups were evaluated using the Mann–Whitney U test for continuous data and the Fisher exact test for categorical data.

**Figure 4.**
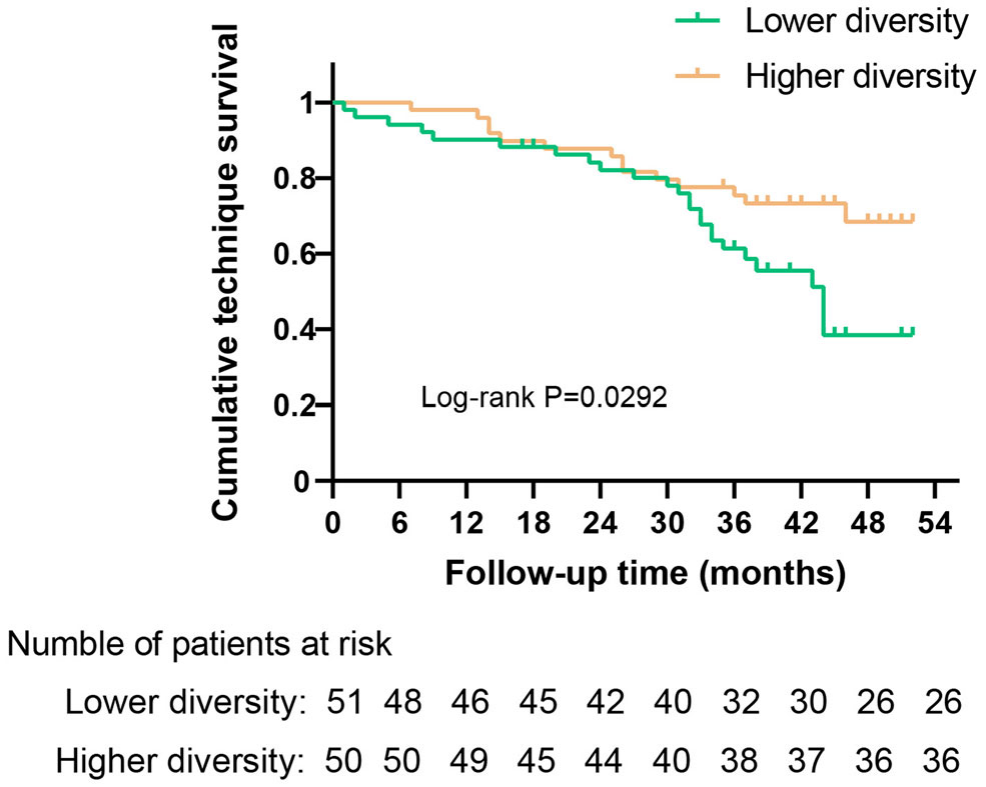
Kaplan–Meier curves of technique failure during follow-up in patients stratified by the median of the inverse Simpson index.

Kaplan–Meier analysis indicated that the cumulative PD technique survival rate was significantly greater in patients with higher diversity than those with lower diversity (*p* = 0.029) (Figure 4). In order to evaluate the association between gut microbial diversity and PD technique failure, we performed univariate Cox proportional hazards analysis showing that lower diversity was significantly associated with a higher risk of technique failure (HR, 2.038; 95% CI, 1.057–3.929; *p* = 0.034). Meanwhile, some variables were also found significantly associated with technique failure, including age, history of coronary heart disease, history of diabetes, history of using statins, albumin, phosphorus, N-terminal pro-brain natriuretic peptide (NT-proBNP) and glucose (*p* < 0.05). Table 4 displayed the HRs and 95% CIs of PD technique failure calculated for all clinical variables. In the multivariate analysis, the same association between lower microbial diversity and shorter technique survival was observed after adjustment for risk factors above. Specifically, PD patients with lower microbial diversity had 1.682-fold increased risk of technique failure compared with higher-diversity patients (HR, 2.682; 95% CI, 1.319–5.456; *p* = 0.006). In addition, older age (HR, 1.034; 95% CI, 1.005–1.063; *p* = 0.020) and the history of diabetes (HR, 5.547; 95% CI, 2.218–13.876; *p* < 0.001) were independent predictors for technique failure in PD patients.

**Table 4.** Univariate and multivariate Cox proportional hazard models for evaluating the relationships between clinical variables and technique failure in PD patients.

Variables	Univariate		Multivariate^a^	
	HR(95% CI)	*p* Value	HR(95% CI)	*p* Value
Age	1.042 (1.016–1.068)	0.001	1.034 (1.005–1.063)	0.020
Sex (women vs. men)	0.952 (0.508–1.784)	0.878		
Systolic BP	1.002 (0.985–1.019)	0.816		
Diastolic BP	0.983 (0.956–1.011)	0.237		
Body mass index	1.050 (0.977–1.128)	0.185		
Dialysis duration	0.999 (0.987–1.010)	0.795		
Urine volume	1.026 (0.605–1.741)	0.923		
Glucose exposure	1.001 (0.993–1.010)	0.746		
Comorbidity^b^				
Hypertension	1.926 (0.263–14.109)	0.519		
Coronary heart disease	5.022 (1.733–14.555)	0.003	1.043 (0.258–4.219)	0.953
Diabetes	4.140 (2.167–7.912)	<0.001	5.547 (2.218–13.876)	<0.001
Cerebral infarction	1.836 (0.768–4.387)	0.172		
Medications^b^				
ACEI	1.367 (0.534–3.498)	0.515		
ARB	0.681 (0.361–1.286)	0.236		
β-Blockers	1.391 (0.729–2.653)	0.316		
α-Blockers	0.828 (0.324–2.121)	0.695		
Antiplatelet drugs	1.167 (0.515–2.647)	0.711		
Statins	1.935 (1.011–3.703)	0.046	1.560 (0.782–3.111)	0.207
Iron supplements	0.958 (0.508–1.805)	0.894		
Phosphate binders	1.060 (0.560–2.007)	0.858		
Prior peritonitis episodes^b^	1.867 (0.823–4.234)	0.135		
Bristol Stool Form Scale	1.003 (0.738–1.363)	0.987		
Number of weekly bowel movements	1.028 (0.933–1.132)	0.576		
Hemoglobin	1.001 (0.986–1.017)	0.873		
Albumin	0.922 (0.864–0.984)	0.014	0.954 (0.882–1.031)	0.234
Prealbumin	0.011 (0–2.260)	0.097		
BUN	1.034 (0.969–1.105)	0.314		
Creatinine	0.999 (0.998–1.000)	0.084		
Uric acid	0.999 (0.995–1.003)	0.634		
Total cholesterol	0.797 (0.595–1.068)	0.129		
Triglyceride	0.814 (0.579–1.145)	0.237		
LDL-C	0.917 (0.667–1.262)	0.595		
HDL-C	0.634 (0.292–1.376)	0.249		
Calcium	0.491 (0.107–2.248)	0.359		
Phosphorus	0.458 (0.210–0.998)	0.049	0.423 (0.146–1.223)	0.112
iPTH	0.998 (0.997–1.000)	0.099		
NT-proBNP	1.000 (1.000–1.000)	0.047	1.000 (1.000–1.000)	0.039
Glucose	1.170 (1.019–1.345)	0.027	0.930 (0.752–1.150)	0.504
Total bilirubin	0.911 (0.789–1.052)	0.204		
ALT	0.972 (0.938–1.007)	0.121		
AST	0.987 (0.950–1.025)	0.493		
ALP	0.997 (0.989–1.004)	0.398		
hsCRP	1.003 (0.981–1.024)	0.814		
nPNA	0.856 (0.235–3.122)	0.814		
Kt/V(total)	0.760 (0.421–1.375)	0.364		
Kt/V(kidney)	0.891 (0.531–1.495)	0.661		
Kt/V(peritoneum)	0.793 (0.369–1.707)	0.554		
Peritoneal transport type				
Low	Reference			
Low average	0.829 (0.185–3.712)	0.806		
High average	1.148 (0.270–4.888)	0.852		
High	0.749 (0.125–4.490)	0.752		
IS	1.001 (0.981–1.021)	0.923		
PCS	1.018 (0.997–1.039)	0.093		
TMAO	1.072 (0.998–1.153)	0.057		
Inverse Simpson index (continuous)	0.967 (0.919–1.016)	0.184		
Inverse Simpson index (categorical)^c^	2.038 (1.057–3.929)	0.034	2.682 (1.319–5.456)	0.006

PD: peritoneal dialysis; BP: blood pressure; ACEI: angiotensin-converting enzyme inhibitors; ARB: angiotensin receptor blockers; BUN: blood urea nitrogen; LDL-C: low-density lipoprotein cholesterol; HDL-C: high-density lipoprotein cholesterol; iPTH: intact parathyroid hormone; NT-proBNP: N-terminal pro-brain natriuretic peptide; ALT: alanine aminotransferase; AST: aspartate aminotransferase; ALP: alkaline phosphatase; hsCRP: high-sensitivity C-reactive protein; nPNA: normalized protein nitrogen appearance rate; IS: indoxyl sulfate; PCS: p-cresyl sulfate; TMAO: trimethylamine N-oxide; CI: confidence interval. ^a^Significant variables (*p* < 0.05) in univariate analyses were added to multivariate Cox proportional hazards model for adjustment. ^b^Patients without comorbidities, history of taking drugs or prior peritonitis episodes were considered as reference. ^c^Patients were stratified by the median of the inverse Simpson index, and the patients in the higher-diversity group were considered as reference.

### Predictive power of the prediction model

**Figure 5.**
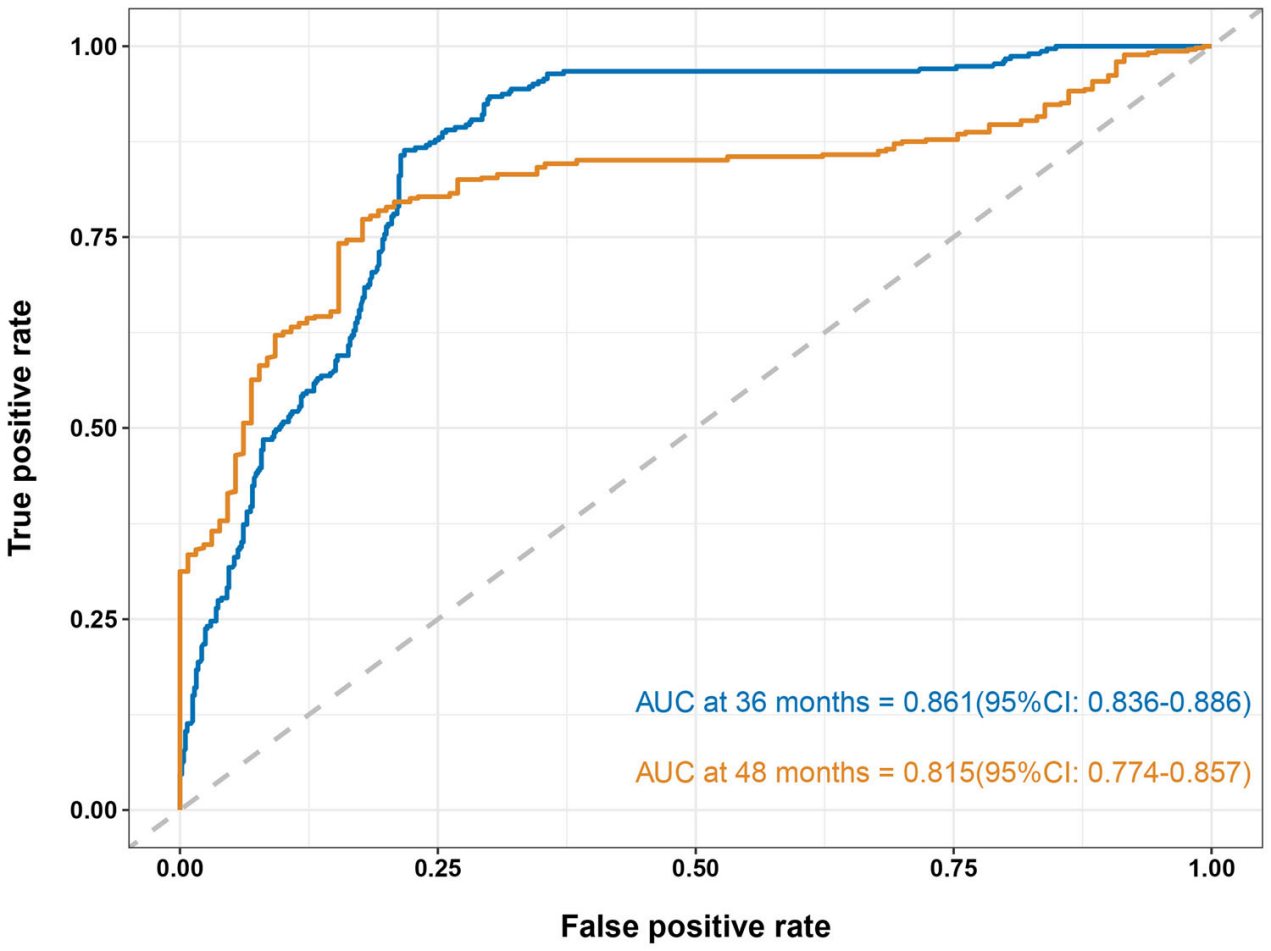
The time-dependent ROC curves of the prediction model for predicting the risk of technique failure in PD patients. ROC: receiver operating characteristic; AUC: area under the curve; CI: confidence interval; PD: peritoneal dialysis.

According to the results from the multivariate Cox proportional hazards model, a prediction model was constructed which contained age, history of diabetes and dichotomous gut microbial diversity. The time-dependent ROC curves were used to assess the predictive power of the new model for technique failure in PD patients. The values of the area under the curve (AUC) were 0.861 (95% CI, 0.836–0.886) at 36 months and 0.815 (95% CI, 0.774–0.857) at 48 months (Figure 5).

## Discussion

In this prospective cohort study, we found that lower diversity of intestinal microbiota was associated with a higher risk of technique failure in PD patients. The association remained significant after adjustment for several potential risk factors, including age, history of coronary heart disease, history of diabetes, history of using statins, albumin, phosphorus, NT-proBNP and glucose. Moreover, a new prediction model which was constructed on the basis of age, history of diabetes and dichotomous alpha diversity performed well in predicting the technique failure of PD patients at 36 and 48 months.

For a large part, the association of lower microbial diversity with poor PD technique survival might be explained by the alterations of gut microbial composition. In this study, we found that Escherichia-Shigella was enriched in PD patients with lower diversity. Escherichia-Shigella is well known as a pro-inflammatory opportunistic pathogen in the intestinal tract, and many studies have found that its relative abundance was positively correlated with the systemic and local levels of pro-inflammatory cytokines, such as interleukin-1β, interleukin-6, CXC chemokine ligand 2 and tumor necrosis factor-2 [33,34]. It also increases intestinal permeability by elevating lipopolysaccharide (LPS) levels in the gut lumen and thereby promotes the translocation of pathogens and harmful metabolites that contribute to systemic inflammatory state [35,36]. The mechanism involved may be NLRP3-dependent signaling pathway [37,38]. In the patients receiving PD treatment, the local and systemic chronic inflammation can stimulate peritoneal angiogenesis and fibrosis [39,40], which results in ultrafiltration failure, increased risk of cardiovascular events, discontinuation of PD therapy or even death [41,42]. Thus, we speculated that the local and systemic chronic inflammatory responses mainly induced by relative expansion of Escherichia-Shigella were important links between lower microbial diversity and higher risk of technique failure of PD patients.

In addition, our findings were consistent with previous studies showing that microbial diversity of PD patients was negatively correlated with triglyceride and positively correlated with HDL-C [43–45]. High triglyceride and low HDL-C are well-established risk factors for cardiovascular diseases [46,47]. This suggested that metabolic disorders might also be an important part in the association of lower microbial diversity with higher risk of PD technique failure. However, there was no direct evidence that elevated triglyceride and reduced HDL-C were associated with PD technique failure in our study, which might be because that our sample size was relatively small. In the meantime, Enterobacteriaceae, which was found to be increased in the lower-diversity group, possesses the genes encoding urease, tryptophanase and p-cresol production enzymes [48] and participates in the generation of several uremic toxins [49,50]. It has been reported that higher level of these toxins could cause disease progression and adverse events through oxidative stress and pro-inflammatory cytokine production [51–53]. In this study, three common gut-derived uremic toxins were measured including IS, PCS and TMAO, but we observed that they were no significantly different between lower- and higher-diversity groups. Part of the possible reason is that most PD patients we included had RKF, while most of the uremic toxins were cleared by the kidney [54], so the excretion of toxins by RKF reverses the effect of Enterobacteriaceae on toxin production. Moreover, it could also not be ruled out that other uremic toxins we did not detect in this study worked.

The results of our study showed that the decrease in gut microbial diversity often meant not only the declines in species richness and evenness, but also the imbalances in the composition and function of these gut microbes including the relatively increase of opportunistic pathogens and loss of beneficial microbes. All these alterations are collectively called gut dysbiosis which may further cause chronic inflammation and metabolic disorders, making the risk of technique failure in PD patients increasing. In fact, gut dysbiosis has been reported to be a potential mechanism for the occurrence and development of many diseases, including inflammatory bowel disease, cancer, cardiovascular diseases, metabolic diseases, kidney diseases and so on [55,56]. As more integrated omics studies are carried out, the role and exact mechanisms of gut dysbiosis in PD technique failure or even other poor outcomes will be gradually revealed.

Our study had several limitations. First, this was a small, single-center cohort, and the therapeutic regimens and compliance of PD patients varied among different PD centers. Therefore, the present results could not extend to all PD patients. Second, compared with 16s ribosomal RNA gene amplicon sequencing that was used to analyze our fecal samples, shotgun metagenomic sequencing provides a more detailed description of gut microbial composition and function. Third, this study was observational in nature, and it could show only correlation but not causation. Finally, we did not assess lifestyles of patients, which might limit the interpretations of our results.

## Conclusion

In conclusion, our study demonstrated that gut microbial diversity was independently correlated with PD technique failure. PD patients with lower gut microbial diversity had a higher risk of technique failure. Future studies with larger scale are needed to verify our findings and to explore whether interventions in some specific microbial taxa may reduce the occurrence of PD technique failure, thereby prolonging the using time of PD and increasing the prevalence of PD.

## Supplementary Material

Supplemental Material
