# Supplementary material for: Association between gut microbial diversity and technique failure in peritoneal dialysis patients
Source: Ren Fail. 2023 Apr 3;45(1):2195014. doi: 10.1080/0886022X.2023.2195014 (PMC10071986; doi:10.1080/0886022X.2023.2195014)
Supplement: Supplemental Material [file IRNF_A_2195014_SM6534.pdf]

Supplementary Table 1. Correlations between the inverse Simpson index and clinical variables.

Variables	Correlation coefficient	P value
Hemoglobin	-0.061	0.542
Albumin	-0.017	0.866
Prealbumin	0.099	0.327
Creatinine	0.029	0.773
Uric acid	-0.027	0.789
BUN	-0.002	0.985
Total cholesterol	-0.037	0.714
Triglyceride	-0.249	0.013
LDL-C	0.005	0.958
HDL-C	0.227	0.024
Calcium	-0.016	0.877
Phosphorus	-0.177	0.077
iPTH	-0.017	0.863
NT-proBNP	0.084	0.402
Glucose	0.013	0.899
Total bilirubin	-0.031	0.758
ALT	0.073	0.466
AST	0.099	0.323
ALP	0.070	0.484
nPNA	0.003	0.980
hsCRP	-0.015	0.880
IS	0.058	0.566
PCS	0.101	0.316
TMAO	0.012	0.902

BUN, blood urea nitrogen; LDL-C, low-density lipoprotein cholesterol; HDL-C, high-density lipoprotein cholesterol; iPTH, intact parathyroid hormone; NT-proBNP, N-terminal pro-brain natriuretic peptide; ALT, alanine aminotransferase; AST, aspartate aminotransferase; ALP, alkaline phosphatase; hsCRP, high-sensitivity C-reactive protein; IS, indoxyl sulfate; PCS, p-cresyl sulfate; TMAO, trimethylamine N-oxide.

The correlation coefficients and P values were analyzed by Spearman rank correlation analysis.

Information Classification: General

Supplementary Table 2. Reasons for PD technique failure.

Reasons for technique failure	Number of patients (n=39)	Lower diversity (≤11.35, n=25)	Higher diversity (>11.35, n=14)	P value
Death (%)	27 (69.2%)	16 (64%)	11 (78.6%)	0.477
Infection (%)	3 (7.7%)	3 (12%)	0 (0%)	0.540
Inadequate dialysis (%)	8 (20.5%)	5 (20%)	3 (21.4%)	1
Mechanical (%)	1 (2.6%)	1 (4%)	0 (0%)	1

PD, peritoneal dialysis.

Data were expressed as n (%). Differences between two groups were evaluated using the Chi-squared test or Fisher exact test as appropriate.

Information Classification: General

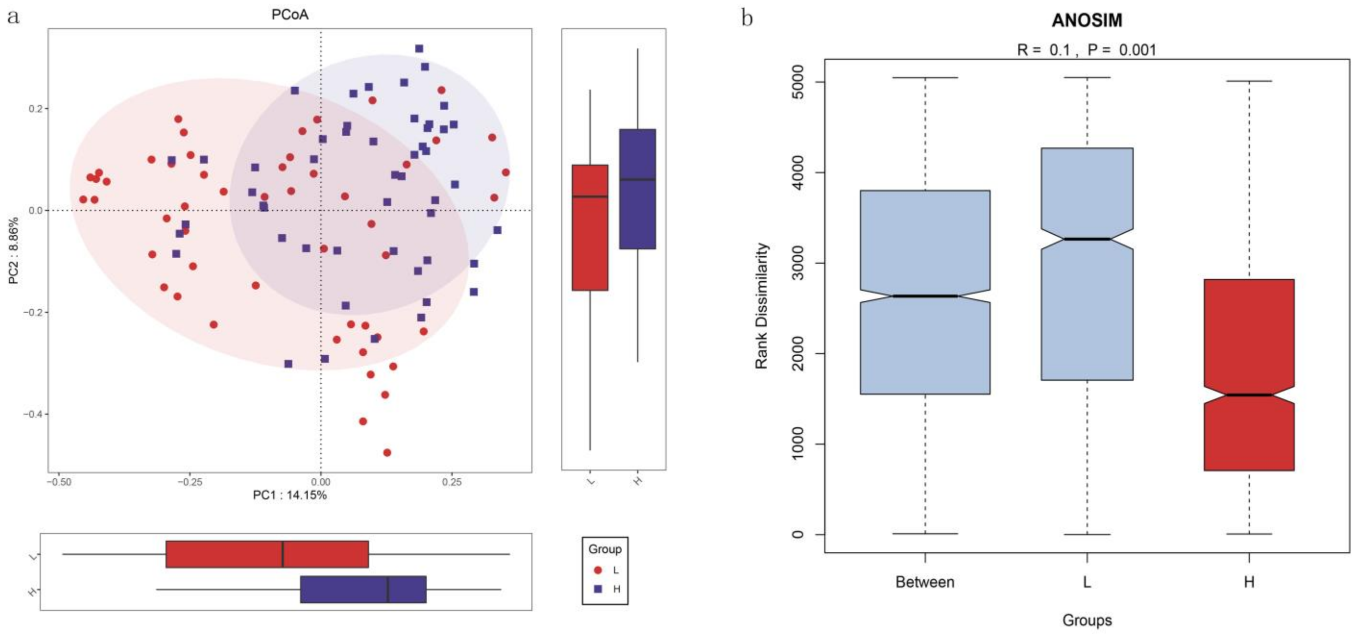

Supplementary Figure 1. Comparison of beta diversity indexes in patients with lower and higher diversity. (a) The PCoA plots of the gut taxonomic composition based on the Bray-Curtis dissimilarity in patients. (b) Analysis of similarities (ANOSIM) based on the Bray-Curtis dissimilarity among groups. PCoA, principal coordinate analysis; L, lower diversity; H, higher diversity.

Information Classification: General
